# Supplementary material for: Point-of-care bulk testing for SARS-CoV-2 by combining hybridization capture with improved colorimetric LAMP
Source: Nat Commun. 2021 Mar 5;12:1467. doi: 10.1038/s41467-021-21627-0 (PMC7935920; doi:10.1038/s41467-021-21627-0)
Supplement: Supplementary file 1 — Supplementary Information [file 41467_2021_21627_MOESM1_ESM.pdf]

Supplementary Data

Point-of-care bulk testing for SARS-CoV-2 by combining hybridization capture with improved colorimetric LAMP (Cap-iLAMP)

Bokelmann et al.

Supplementary Table 1: Sequences of oligonucleotides used in this study.

Oligonucleotide	Sequence (5‘ -> 3‘)	Supplier	Purification	Reference
CV1 (F3)	TCCAGATGAGGATGAAGAAGA	Sigma Aldrich	Desalted	Lamb et al (2020)
CV2 (B3)	AGTCTGAACAACTGGTGTAAG	Sigma Aldrich	Desalted	Lamb et al (2020)
CV3 (FIP)	AGAGCAGCAGAAGTGGCACAGGTGA TTGTGAAGAAGAAGAG	Sigma Aldrich	Desalted	Lamb et al (2020)
CV4 (BIP)	TCAACCTGAAGAAGAGCAAGAACTGA TTGTCCTCACTGCC	Sigma Aldrich	Desalted	Lamb et al (2020)
CV5 (LF)	CTCATATTGAGTTGATGGCTCA	Sigma Aldrich	Desalted	Lamb et al (2020)
CV6 (LB)	ACAAACTGTTGGTCAACAAGAC	Sigma Aldrich	Desalted	Lamb et al (2020)
CV9 (F3)	CTGCACCTCATGGTCATGTT	Sigma Aldrich	Desalted	Zhang et al. (2020)
CV10 (B3)	AGCTCGTCGCCTAAGTCAA	Sigma Aldrich	Desalted	Zhang et al. (2020)
CV11 (FIP)	GAGGGACAAGGACACCAAGTGTATG GTTGAGCTGGTAGCAGA	Sigma Aldrich	Desalted	Zhang et al. (2020)
CV12 (BIP)	CCAGTGGCTTACCGCAAGGTTTTAGA TCGGCGCCGTAAC	Sigma Aldrich	Desalted	Zhang et al. (2020)
CV13 (LF)	CCGTACTGAATGCCTTCGAGT	Sigma Aldrich	Desalted	Zhang et al. (2020)
CV14 (LB)	TTCGTAAGAACGGTAATAAAGGAGC	Sigma Aldrich	Desalted	Zhang et al. (2020)
CV15 (F3)	TGGCTACTACCGAAGAGCT	Sigma Aldrich	Desalted	Zhang et al. (2020)
CV16 (B3)	TGCAGCATTGTTAGCAGGAT	Sigma Aldrich	Desalted	Zhang et al. (2020)
CV17 (FIP)	TCTGGCCCAGTTCCTAGGTAGTCCAG ACGAATTCGTGGTGG	Sigma Aldrich	Desalted	Zhang et al. (2020)
CV18 (BIP)	AGACGGCATCATATGGGTTGCACGGG TGCCAATGTGATCT	Sigma Aldrich	Desalted	Zhang et al. (2020)
CV19 (LF)	GGACTGAGATCTTTCATTTTACCGT	Sigma Aldrich	Desalted	Zhang et al. (2020)
CV20 (LB)	ACTGAGGGAGCCTTGAATACA	Sigma Aldrich	Desalted	Zhang et al. (2020)
CV2_btn (capture oligonucleotide)	/BtnTg/TTAAATAACCACTAAAACTATTC ACTTCAATAGTCTGAACAACTGGTGTA AG	Sigma Aldrich	Desalted	This study
CV16_btn (capture oligonucleotide)	/BtnTg/AATGTTGTTCCTTGAGGAAGTT GTAGCACGATTGCAGCATTGTTAGCA GGAT	Sigma Aldrich	Desalted	This study
E_Sarbeco_R2_bt n (capture oligonucleotide)	/BtnTg/AAAGAAGGTTTTACAAGACTCA CGTTAACAATATTGCAGCAGTACGCA CACA	Sigma Aldrich	Desalted	This study

Supplementary Table 2: Price calculation of Cap-iLAMP tests.

Reagent	Order unit volume	Order unit cost [€]	Volume/reaction [µl]	Cost /reaction [€]
iLAMP reagents				
WarmStart Colorimetric LAMP Master Mix (2x)	6.25ml	673.9	15.0	1.6
Primer mix (10x)	6x200nmol	58.1	3.0	< 1 cent
ATP (100mM)	250µl	28.9	0.3	< 1 cent
SYTO-9 (100µM)	100ul (5mM)	408.0	0.3 of 1/200	< 1 cent
Tte UvrD helicase (20ng/µl)	25µl	56.2	0.2	0.3
Protector RNase inhibitor (40U/µl)	250µl	502.4	0.3	0.6
Thermostable incorganic pyrophosphatase (2U/µl)	625µl	227.8	0.8	0.3
SYBR green I	1ml	640.8	0.5	0.3
Total iLAMP cost				3.2
Capture reagents				
Dynabeads MyOne Streptavidin C1	10ml	1364.1	20.0	2.7
Biotinylated capture oligonucleotide	250nmole	120.5	0.004nmol	< 1 cent
Total capture cost				2.7
Twist SARS-CoV-2 RNA positive control	100µl	416.0	< 0.1µl	< 1 cent
Optional: Twist SARS-CoV-2 RNA for capture inhibition control	100µl	416.0	0.3	1.0
Total cost 2 captures, 8 iLAMPs (4x Orf1a gene, 4x N gene)				**31.1**
Cost per individual in pool of 26				**1.2**
Optional:Total cost 3 captures, 10 iLAMPs (5x Orf1a gene, 5x N gene)				41.3
Cost per individual in pool of 26, including capture inhibition control				1.6

Supplementary Table 3: Cap-iLAMP results of individual SARS-CoV-2 positive samples. The sample ID, the Ct value obtained via RT-qPCR assay targeting the SARS-CoV-2 *E* gene, hue of two repeated experiments for the *Orf1a* gene assay and *N* gene assay, diagnostic result, and predictive agreement are stated. Assays with a hue >28.5° (yellow background) or hue ≤28.5° (rose background) are considered positive or negative, respectively. A red dotted line is used to indicate samples with high viral loads (Ct < 24) and low viral loads (Ct ≥24). Samples with Ct < 24 are likely highly infectious. The samples used for the 2nd experiment underwent an additional freeze/thaw cycle.

ID	Ct (RT-qPCR)	Hue [°] 1st experiment		Hue [°] 2nd experiment		RESULT	Predictive agreement
		Orf1a	N	Orf1a	N		
P1	12.2	46.50	46.50	46.50	47.50	SARS-CoV-2 positive	yes
P2	13	47.50	45.50	46.50	46.00	SARS-CoV-2 positive	yes
P3	13.8	47.00	46.50	44.00	45.50	SARS-CoV-2 positive	yes
P4	16.4	45.00	46.50	44.50	47.50	SARS-CoV-2 positive	yes
P5	17	23.00	25.50	18	47.00	presumptive positive	yes
P6	17.3	43.50	46.00	45.00	45.00	SARS-CoV-2 positive	yes
P7	18.8	45.00	46.00	45.50	45.00	SARS-CoV-2 positive	yes
P8	18.9	45.50	48.50	46.50	46.00	SARS-CoV-2 positive	yes
P9	19	46.00	46.00	46.50	48.50	SARS-CoV-2 positive	yes
P10	19	45.00	46.00	45.00	47.50	SARS-CoV-2 positive	yes
P11	19.2	48.00	47.50	46.00	45.00	SARS-CoV-2 positive	yes
P12	19.8	47.00	47.00	48.00	49.50	SARS-CoV-2 positive	yes
P13	20.1	46.00	47.00	27.00	45.50	SARS-CoV-2 positive	yes
P14	20.3	43.50	47.00	46.50	46.50	SARS-CoV-2 positive	yes
P31	22	39.50	48.50	40.00	41.00	SARS-CoV-2 positive	yes
P15	22.1	47.00	47.50	46.50	37.50	SARS-CoV-2 positive	yes
P16	22.1	45.00	45.50	45.00	44.50	SARS-CoV-2 positive	yes
ring 59	22.5	44	45.5			SARS-CoV-2 positive	yes
ring 63	22.5	39.5	40			SARS-CoV-2 positive	yes
P17	22.8	46.50	46.50	44.50	46.50	SARS-CoV-2 positive	yes
P18	23.1	23.50	43.50	25	27.00	presumptive positive	yes
P19	23.6	44.50	45.00	40.00	47.50	SARS-CoV-2 positive	yes
P20	24.2	24.50	47.00	19	25.5	presumptive positive	yes
P21	24.2	45.00	34.50	25.5	26	SARS-CoV-2 positive	yes
P22	24.4	45.00	47.00	26	42.50	SARS-CoV-2 positive	yes
P23	25	19.00	25.00	18	20.5	SARS-CoV-2 negative	no
P24	25	25.50	47.00	13.5	13.5	presumptive positive	yes
P25	25	25.00	30.00	26	28	presumptive positive	yes
P26	25.2	33.50	45.50	24.5	28	SARS-CoV-2 positive	yes
ring 64	26.5	37.5	40			SARS-CoV-2 positive	yes
P27	28.2	22.50	28.50	43.00	29	SARS-CoV-2 positive	yes
P28	28.5	42.50	44.50	44.50	22.5	SARS-CoV-2 positive	yes
ring 61	29.5	38	39			SARS-CoV-2 positive	yes
P29	30.6	23.50	30.00	42.50	28	SARS-CoV-2 positive	yes
P30	32.2	25.00	29.00	19.5	24	presumptive positive	yes

Supplementary Table 4: Cap-iLAMP results of pooled SARS-CoV-2 positive samples. The spike-in sample ID, the Ct value of the spike-in samples obtained via RT-qPCR assay targeting the SARS-CoV-2 *E* gene, hue of the *Orf1a* gene assay and *N* gene assay, diagnostic result, and predictive agreement are stated. Assays with a hue >28.5° (yellow background) or hue ≤28.5° (rose background) are considered positive or negative, respectively. A red dotted line indicates the separation between samples with high viral loads (Ct < 24) and low viral loads (Ct ≥24). Samples with Ct < 24 are likely highly infectious.

Spike-in ID (1/26 of pool)	Spike-in Ct (RT-qPCR)	Hue [°]		RESULT	Predictive agreement
		Orf1a	N		
P1	12.2	47.5	48.0	SARS-CoV-2 positive	yes
P3	13.8	47.0	48.5	SARS-CoV-2 positive	yes
P6	17.3	46.5	46.0	SARS-CoV-2 positive	yes
P8	18.9	25.5	29.0	presumptive positive	yes
P12	19.8	23.5	45.5	presumptive positive	yes
P15	22.1	24.0	44.5	presumptive positive	yes
P17	22.8	22.0	47.0	presumptive positive	yes
P20	24.2	20.0	25.5	SARS-CoV-2 negative	no
P-A	24.6	51.5	55.5	SARS-CoV-2 positive	yes
P-B	24.7	49.5	56.5	SARS-CoV-2 positive	yes
P24	25.0	27.0	45.5	presumptive positive	yes
P26	25.2	27.5	31.0	presumptive positive	yes
P-C	25.4	54.5	55.5	SARS-CoV-2 positive	yes
P28	28.5	26.5	31.0	presumptive positive	yes
P29	30.6	26.5	31.0	presumptive positive	yes
P30	32.2	25.5	30.0	presumptive positive	yes
P32	33.0	24.5	26.5	SARS-CoV-2 negative	no
P33	36.0	22.0	26.5	SARS-CoV-2 negative	no

Supplementary Table 5: Evaluation of Cap-iLAMP results. A conservative assignment to predict SARS-CoV-2 infection requires both assays targeting the *Orf1a* and the *N* gene to be positive, while a relaxed assignment requires at least one assay to be positive. For each of the two assays, a water negative control as well as a sample-specific positive/inhibition control comprised of sample and artificial viral RNA can be included as quality control.

Assay	Patient sample	+ Inhibition control	Negative control	RESULT
Orf1a	+	+	-	SARS-CoV-2-positive (conservative assignment)
N	+	+	-	
Orf1a	-	+	-	Presumptive positive (relaxed assignment)
N	+	+	-	
Orf1a	+	+	-	
N	-	+	-	
Orf1a	-	+	-	SARS-CoV-2-negative
N	-	+	-	
Orf1a	+	+	+	QC failure
N	+	+	+	
Orf1a	-	-	-	
N	-	-	-	

Supplementary Table 6: Diagnostic predictive power of Cap-iLAMP. A conservative assignment to predict SARS-CoV-2 infection requires both assays targeting the *Orf1a* and the *N* gene to be positive, while a relaxed assignment requires at least one assay to be positive. Predictive agreements are compared and ranked optimal (green), sub-optimal (light green), critical (orange) or striped if in between. False positive/negative rate and negative/positive predictive agreement are stated together with the 95% binomial confidence interval. The asterisk indicates the false positive rate and predictive power, when false positive amplification of N475 and N476 from Supplementary Figure 8 are attributed to experimental carry-over from the adjacent positive sample P31.

	**Conservative assignment**		**Relaxed assignment**	
SARS-CoV-2 negative samples	*False positive rate*	*Negative predictive agreement*	*False positive rate*	*Negative predictive agreement*
All (n=236)	0% (0/236. 0-1.3%)	100% (236/236. 98.7-100%)	1.3% (3/236. 0.3-3.7%)<br>0.4%* (1/236. 0-2.3%)	98.7% (233/236. 96.3-99.7%)<br>99.6%* (235/236. 97.7-100%)
SARS-CoV-2 positive samples	*False negative rate*	*Positive predictive agreement*	*False negative rate*	*Positive predictive agreement*
Infectious (Ct<24) (n=22)	9.1% (2/22. 1.1-29.2%)	90.9% (20/22. 70.8-98.9%)	0% (0/22. 0-15.4%)	100% (22/22. 84.6-100%)
All (Ct 12.2 – 32.2) (n=35)	20% (7/35. 8.4-36.9%)	80% (28/35. 63.1-91.6%)	2.9% (1/35. 0.1-14.9%)	97.1% (34/35. 85.1-99.9%)
Pools. 1/26 SARS-CoV-2 positive sample	*False negative rate*	*Positive predictive agreement*	*False negative rate*	*Positive predictive agreement*
Infectious (Ct<24) (n=7)	57.1% (4/7. 18.4-90.1%)	42.9% (3/7. 9.9-81.6%)	0% (0/7. 0-41%)	100% (7/7. 59-100%)
All (Ct 12.2 – 36) (n=18)	66.7% (12/18. 41-86.7%)	33.3% (6/18. 13.3-59%)	16.7% (3/18. 1.4-34.7%)	83.3% (15/18. CI 65.3-98.6%)

Supplementary Table 7: Comparison of sensitivity and specificity of point-of-care tests for SARS-CoV-2. The test target (antigen or RNA), name, number of evaluated studies, sample number, false negative rate [%], average sensitivity (positive predictive agreement) [%], false positive rate [%], average specificity (negative predictive agreement) [%], as well as the respective 95% binomial confidence interval is stated. Data from several studies was evaluated in a meta-analysis by Dinnes et al. 2020. Data of Cap-iLAMP is given for the relaxed assignment. Performance is compared and ranked optimal (green), sub-optimal (light green), critical (orange) or striped if in between. Both the Xpert Xpress and ID NOW test can only be used with proprietary analysis machines and sample cartridges. The asterisk indicates the values, when false positive amplification of N475 and N476 from Supplementary Figure 8 are attributed to experimental carry-over from the adjacent positive sample P31. Antigen test types are either fluorescence immunoassays (FIA) or colloidal gold-based immunoassays (CGIA).

	Test name	Test type	Studies	Total Samples	Positive samples	False negative rate	Average SENSITIVITY	False positive rate	Average SPECIFICITY	Reference
Antigen	Coris Bioconcept	CGIA	2	466	226	45.6 (39.2-52.1)	54.4 (47.9-60.8)	0.4 (0.1-2.3)	99.6 (97.7-99.9)	*Dinnes et al. 2020*
Antigen	Liming	CGIA	1	19	9	100 (66.4-100)	0 (0-33.6)	10 (0.3-44.5)	90.0 (55.5-99.7)	*Dinnes et al. 2020*
Antigen	RapiGEN	CGIA	1	109	79	38 (27.3-49.6)	62.0 (50.4-72.7)	0 (0-11.6)	100 (88.4-100)	*Dinnes et al. 2020*
Antigen	Beijing Savant	FIA	1	109	78	83.3 (73.2-90.8)	16.7 (9.2-26.8)	0 (0-11.2)	100 (88.8-100)	*Dinnes et al. 2020*
Antigen	Shenzhen Bioeasy	FIA	2	238	162	10.5 (6.6-16.2)	89.5 (83.8-93.3)	0 (0-4.8)	100 (95.2-100)	*Dinnes et al. 2020*
Antigen	In-house	FIA	1	239	208	32.2 (25.9-39)	67.8 (61.0-74.1)	0 (0-11.2)	100 (88.8-100)	*Dinnes et al. 2020*
RNA	Xpert Xpress (GeneXpert machine)	RT-PCR	6	919	479	0.6 (0.2-2)	99.4 (98.0-99.8)	3.2 (1-9.4)	96.8 (90.6-99)	*Dinnes et al. 2020*
RNA	ID NOW (ID NOW machine)	isothermal amplification	5	1003	496	23.2 (19.7-27.1)	76.8 (72.9-80.3)	0.4 (0.1-1.6)	99.6 (98.4-99.9)	*Dinnes et al. 2020*
RNA	Cap-iLAMP	isothermal amplification	1	271	35	2.9 (0.1-14.9)	97.1 (85.1-99.9)	1.3 (0.3-3.7) or 0.4* (0-2.3)	98.7 (96.3-99.7) or 99.6* (97.7-100)	this study

Supplementary Table 8: Composition of a 10x LAMP primer master mix.

**Component**	**V/µl**	**Final concentration**
F3 primer (100µM)	8	2µM
B3 primer (100µM)	8	2µM
FIP primer (100µM)	64	16µM
BIP primer (100µM)	64	16µM
LF primer (100µM)	16	4µM
LB primer (100µM)	16	4µM
Water	224	-
**Total**	**400**	

Supplementary Table 9: Composition of the iLAMP master mix for a 30µl reaction.

**Component**	**V/µl**	**Concentration in 30µl reaction**
WarmStart® Colorimetric LAMP Master Mix (2x) (NEB)	15	1x
Primer mix (10x)	3	1x
ATP (100mM)	0.3	1mM
SYTO-9 (100µM)	0.3	1µM
Tte UvrD helicase (20ng/µl)	0.15	0.1ng/µl
Protector RNase inhibitor (40U/µl) (Sigma Aldrich)	0.3	0.4U/µl
Thermostable incorganic pyrophosphatase (2U/µl) (NEB)	0.75	0.05U/µl
Nuclease free water	0.2	-
**Total**	**20**	

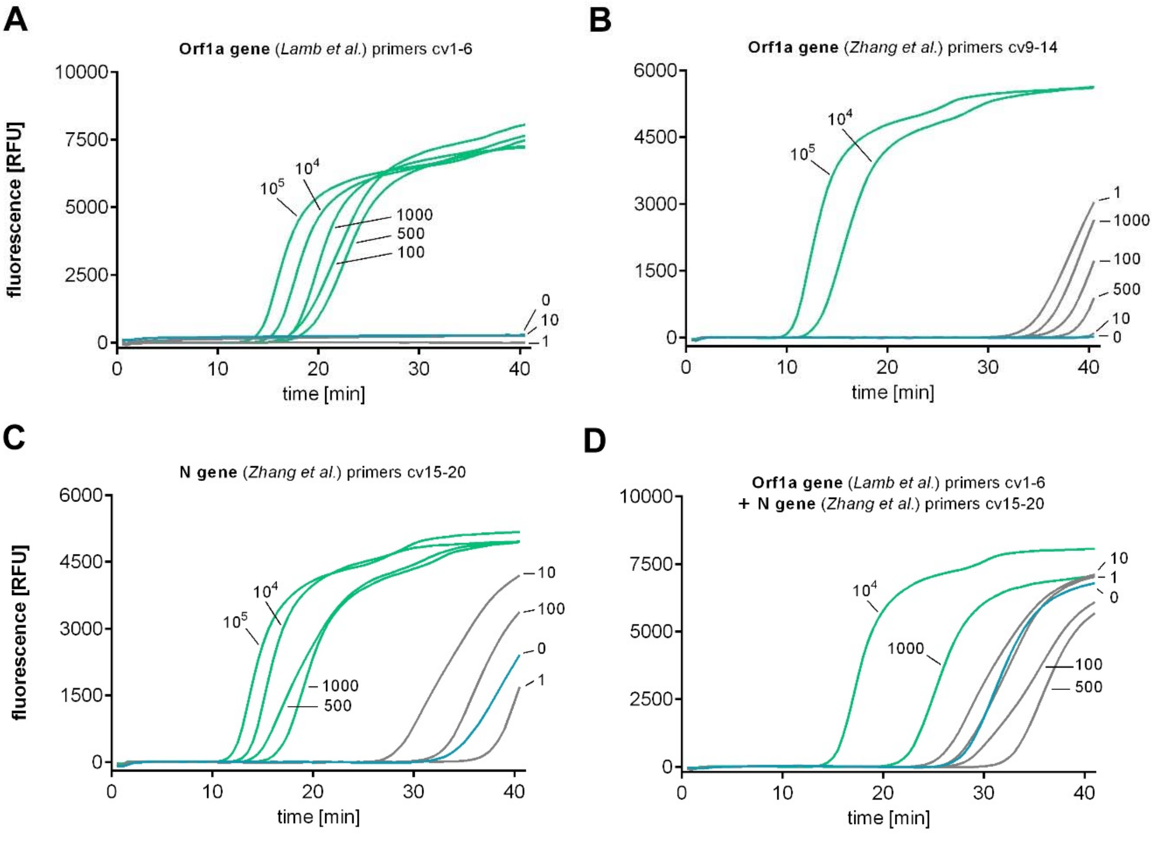

Supplementary Fig. 1. Sensitivity of different primer sets targeting SARS-CoV-2 RNA. (A) Amplification curves of LAMP reactions using primers CV1-6 and various copy numbers of artificial SARS-CoV-2 RNA. (B) Amplification curves of LAMP reactions using primers CV9-14 and various copy numbers of artificial SARS-CoV-2 RNA. (C) Amplification curves of LAMP reactions using primers CV15-20 and various copy numbers of artificial SARS-CoV-2 RNA. (D) Amplification curves of LAMP reactions using two primer sets (CV1-6 + CV15-20) and various copy numbers of artificial SARS-CoV-2 RNA. Amplification proceeded at 65°C. Source data are provided as a Source Data file.

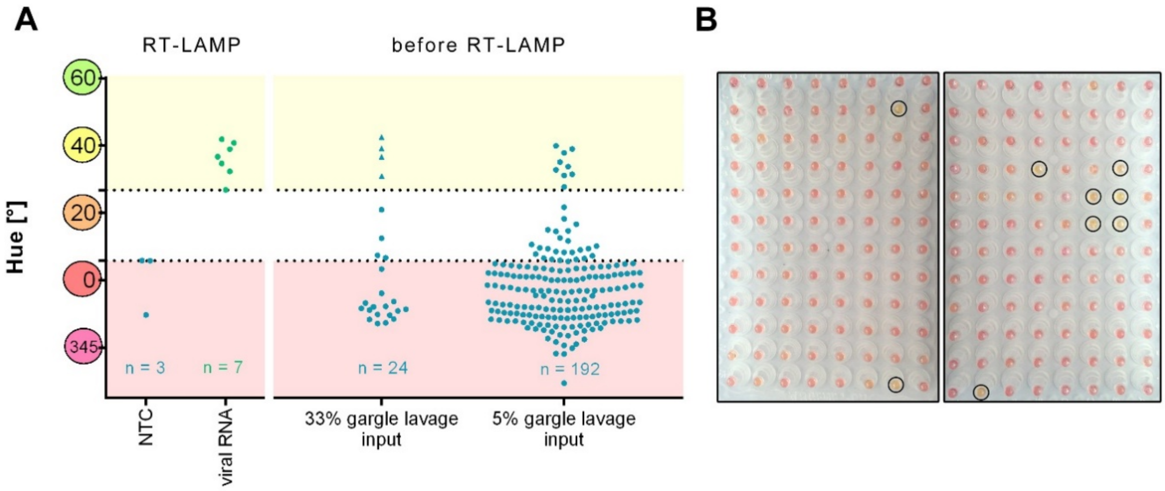

Supplementary Fig. 2. pH variability in diagnostic samples can lead to false positives. (A) Hues measured for negative and positive control reactions after RT-LAMP (left) and patient gargle lavage (GL) samples added directly to the LAMP reaction in different ratios (33% and 5%) before performing RT-LAMP. Triangles mark nasopharyngeal swab eluate samples. (B) LAMP reaction mixes including directly added gargle lavage samples before RT-LAMP. Samples passing the positive hue threshold are marked by circles. Source data are provided as a Source Data file.

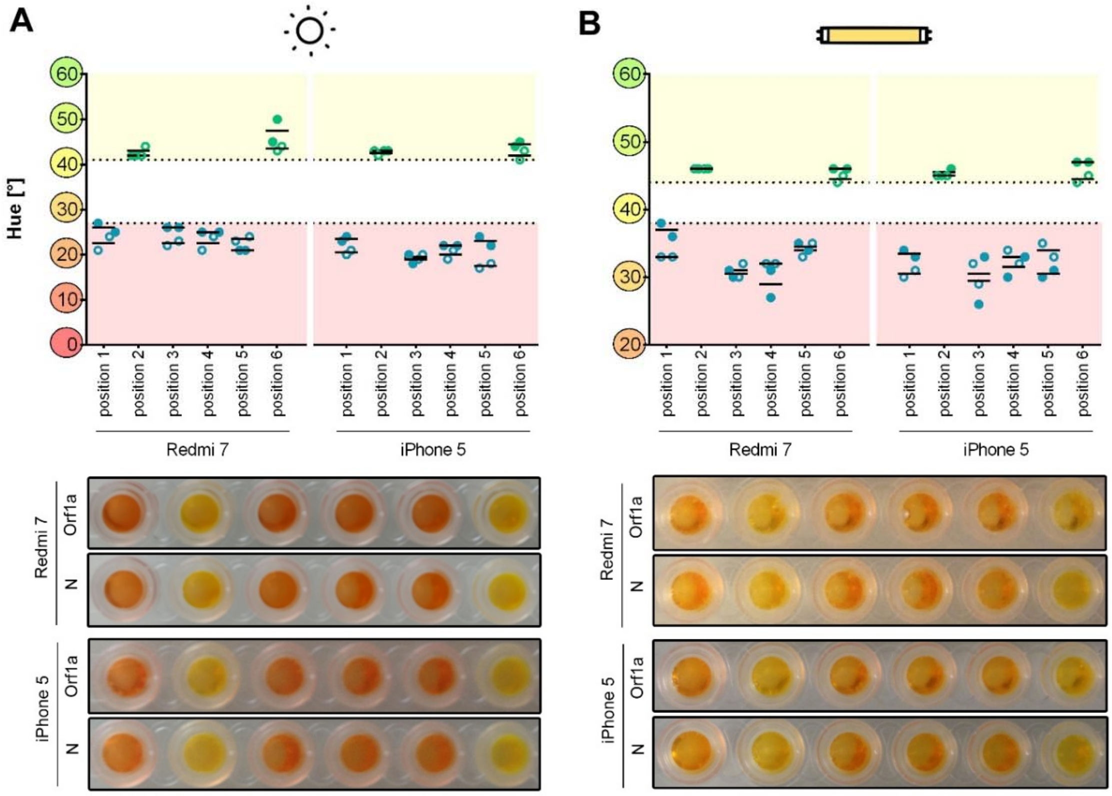

Supplementary Fig. 3: Influence of different smartphone models and light conditions on color scoring. Photographs were taken using Redmi 7 model M1810F6LG and iPhone 5 model 1429. Solid circles indicate values of the *Orf1a* assay while hollow circles denote values of the *N* gene assay. Samples negative for SARS-CoV-2 (position 1, 3, 4 and 5) are shown in blue while SARS-CoV-2 positive samples (position 2 and 6) are depicted in green. Maximum separation of negative and positive samples is shown by dotted lines. (A) Smartphone photographs of Cap-iLAMP reactions obtained under daylight and corresponding hue values measured in duplicates. (B) Smartphone photographs of Cap-iLAMP reactions obtained under fluorescent light (Philips tube light 54W/830/HO) and corresponding hue values measured in duplicates. Source data are provided as a Source Data file.

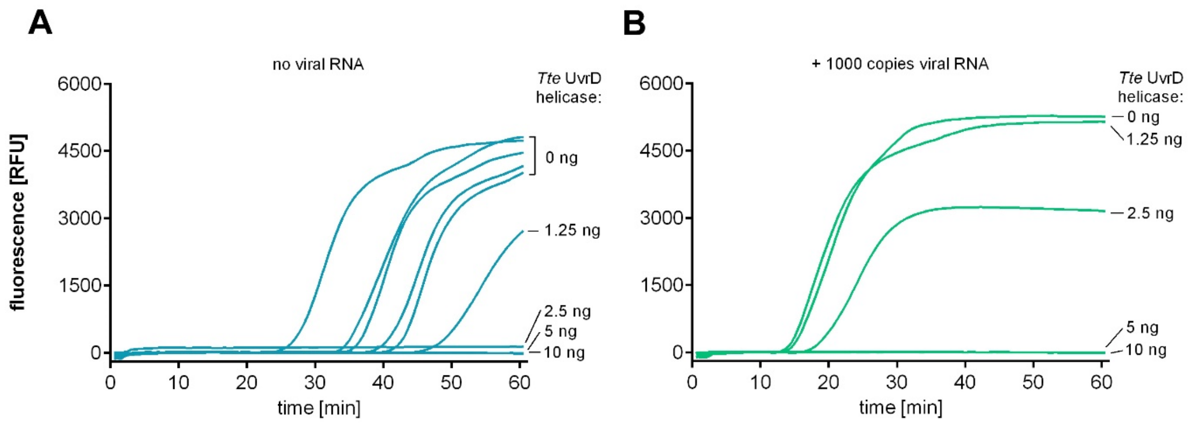

Supplementary Fig. 4. Evaluation of different amounts of *Tte* UvrD helicase in LAMP reactions. (A) Amplification plots of negative controls and (B) positive controls containing 1000 copies of artificial SARS-CoV-2 RNA. Amount of *Tte* UvrD helicase in 20µl reaction are indicated on the right. The primer set used targeted the *N* gene (CV15-20), the assay proceeded at 65°C. Source data are provided as a Source Data file.

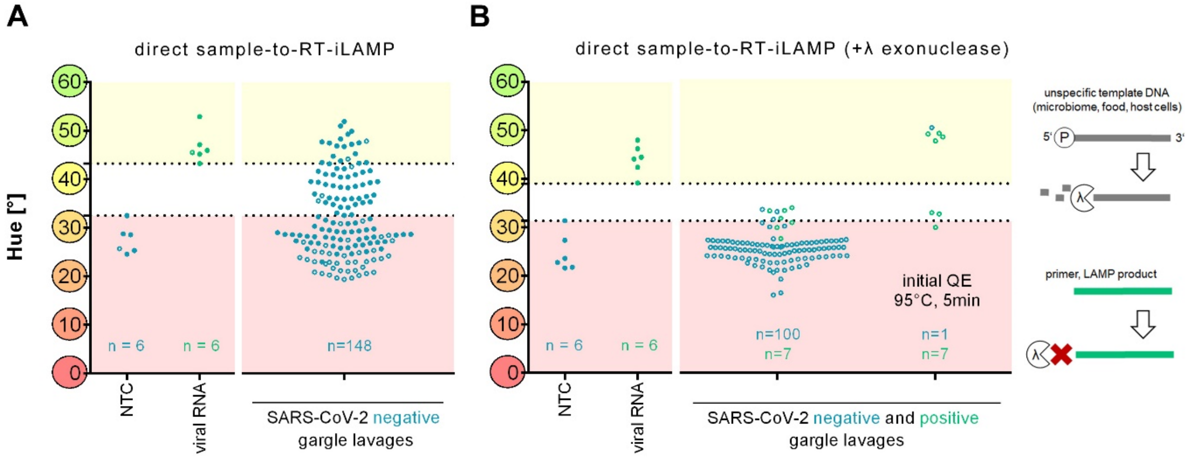

Supplementary Fig. 5. Direct LAMP of patient gargle lavage samples can lead to false positives and false negatives. (A) Hues measured for negative (n=6) and positive (n=6) controls after RT-iLAMP reactions (left side) and after RT-iLAMP of SARS-CoV-2-negative (n=148) gargle lavage samples directly added to the reaction mix. (B) Hues measured after RT-iLAMP reactions preceeded by a λ exonuclease (NEB) digestion step (5U per reaction for 10-15min at 25°C) for negative (n=6) and positive (n=6) controls (left side), SARS-CoV-2-negative (n=100) and SARS-CoV-2-positive (n=7) gargle lavage samples directly added to the reaction mix (middle) and SARS-CoV-2-negative (n=1) and SARS-CoV-2-positive (n=7) gargle lavage samples directly added to the reaction mix digested with QuickExtract (QE) and heat inactivated for 5min at 95°C before LAMP (right). Schematic drawing of λ exonuclease digestion shown on the right. Primers and LAMP reaction products do not possess a 5'-phosphate and are thus not a target for λ exonuclease digestion. All reactions contained 10µg T4 gene 32 protein (NEB) as it could alleviate inhibition of some gargle lavages in initial experiments. Source data are provided as a Source Data file.

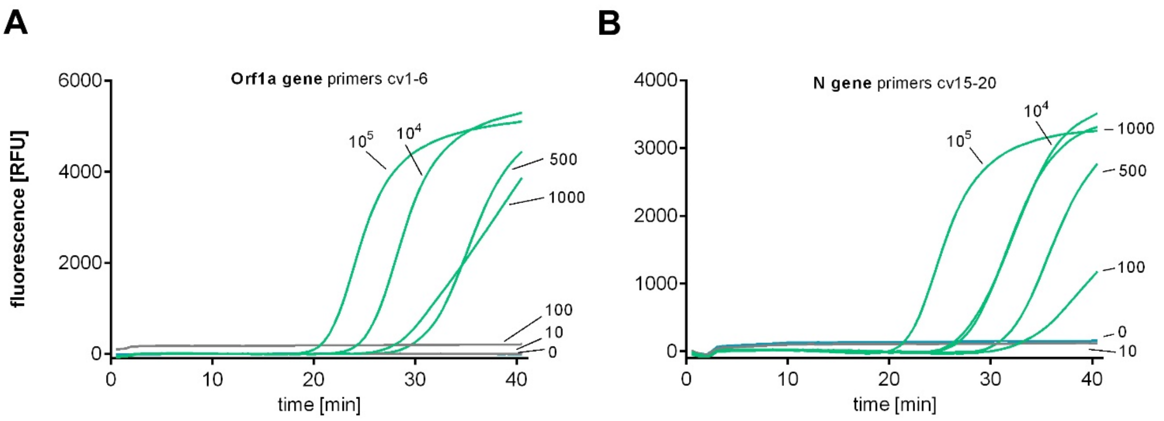

Supplementary Fig. 6. Sensitivity of different primer sets targeting SARS-CoV-2 RNA in the final RT-iLAMP formulation. (A) Amplification curves of LAMP reactions targeting the *Orf1a* gene (primers CV1-6) and various copy numbers of artificial SARS-CoV-2 RNA. (B) Amplification curves of LAMP reactions targeting the *N* gene (CV15-20) and various copy numbers of artificial SARS-CoV-2 RNA. The 30µl iLAMP reaction contained 10µl capture elution buffer. Amplification proceeded at 65°C. Source data are provided as a Source Data file.

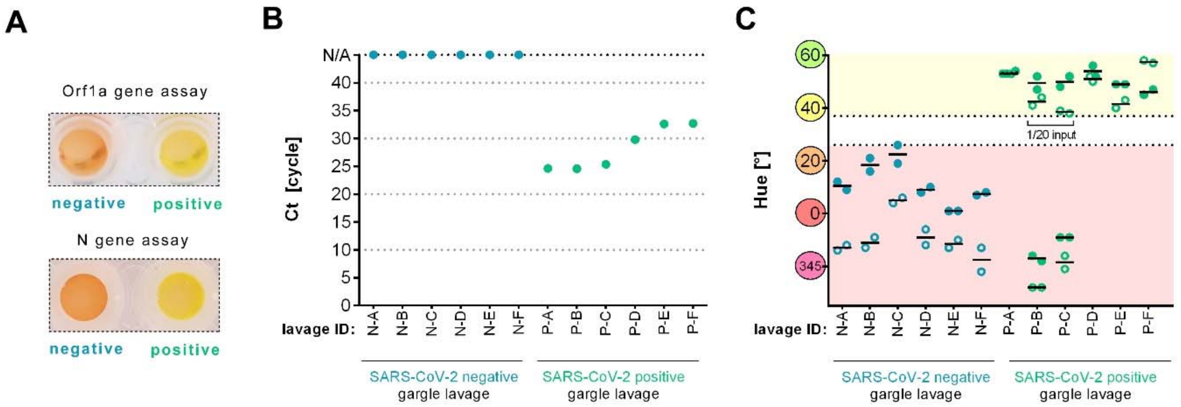

Supplementary Fig. 7: Detection of SARS-CoV-2 in gargle lavage samples with Cap-iLAMP using only two wash steps after capture. (A) Color of negative and positive control after Cap-iLAMP targeting the *Orf1a* and *N* gene of SARS-CoV-2. (B) Ct values of individual patient gargle lavage samples obtained via RT-qPCR assay targeting the SARS-CoV-2 *E* gene. (C) Hue of individual gargle lavage samples after Cap-iLAMP measured in duplicates. Solid circles indicate values of the *Orf1a* assay while hollow circles denote values of the *N* gene assay. Data obtained from healthy individuals are shown in blue while SARS-CoV-2 positive patient samples are depicted in green. Two positive samples (P-B and P-C) are negative for both assays, but are positive when only 1/20 of input is used thus hinting to inhibition by these samples if not diluted. Source data are provided as a Source Data file.

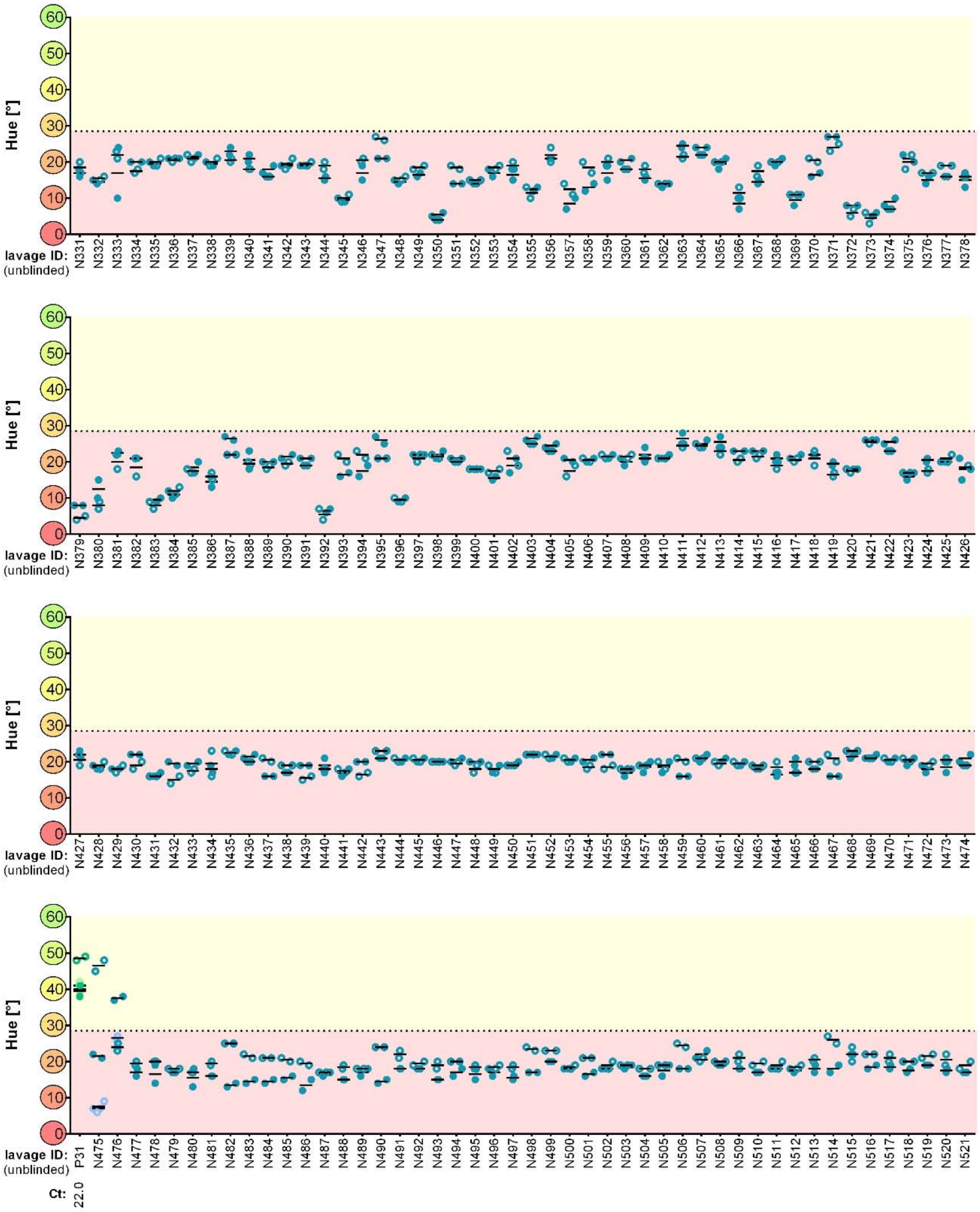

Supplementary Fig. 8. Blinded detection of a single SARS-CoV-2 positive gargle lavage out of 192 gargle lavage samples. Hue of individual gargle lavage samples after Cap-iLAMP measured in duplicates. Data obtained from healthy individuals are shown in blue while the single SARS-CoV-2 positive patient sample is depicted in green. The Ct value of this sample obtained via RT-qPCR assay targeting the SARS-CoV-2 *E* gene is stated. Solid circles indicate values of the *Orf1a* assay while hollow circles denote values of the *N* gene assay. Assays with a hue >28.5° (dotted line) are considered positive. Suspiciously, the two SARS-CoV-2 negative samples that were positive for one assay were located in wells adjacent to the true positive sample in the plate containing the capture eluates and thus could be due to experimental carry-over. Repeating the assays for these samples resulted in correct negative assignment for both gene assays. Repeated experiments are depicted in light color and respective samples underwent an additional freeze/thaw cycle. Source data are provided as a Source Data file.
