# Supplementary material for: Point-of-care bulk testing for SARS-CoV-2 by combining hybridization capture with improved colorimetric LAMP
Source: Nat Commun. 2021 Mar 5;12:1467. doi: 10.1038/s41467-021-21627-0 (PMC7935920; doi:10.1038/s41467-021-21627-0)
Supplement: Supplementary file 3 — Reporting Summary [file 41467_2021_21627_MOESM3_ESM.pdf]

nature research

Corresponding author(s): Stephan Riesenberg

Last updated by author(s): Jan 28, 2021

Reporting Summary

Nature Research wishes to improve the reproducibility of the work that we publish. This form provides structure for consistency and transparency in reporting. For further information on Nature Research policies, see our Editorial Policies and the Editorial Policy Checklist.

Statistics

For all statistical analyses, confirm that the following items are present in the figure legend, table legend, main text, or Methods section.

n/a	Confirmed	
☐	☒	The exact sample size (*n*) for each experimental group/condition, given as a discrete number and unit of measurement
☒	☐	A statement on whether measurements were taken from distinct samples or whether the same sample was measured repeatedly
☒	☐	The statistical test(s) used AND whether they are one- or two-sided<br>*Only common tests should be described solely by name; describe more complex techniques in the Methods section.*
☒	☐	A description of all covariates tested
☒	☐	A description of any assumptions or corrections, such as tests of normality and adjustment for multiple comparisons
☒	☐	A full description of the statistical parameters including central tendency (e.g. means) or other basic estimates (e.g. regression coefficient) AND variation (e.g. standard deviation) or associated estimates of uncertainty (e.g. confidence intervals)
☒	☐	For null hypothesis testing, the test statistic (e.g. *F*, *t*, *r*) with confidence intervals, effect sizes, degrees of freedom and *P* value noted<br>*Give P values as exact values whenever suitable.*
☒	☐	For Bayesian analysis, information on the choice of priors and Markov chain Monte Carlo settings
☒	☐	For hierarchical and complex designs, identification of the appropriate level for tests and full reporting of outcomes
☒	☐	Estimates of effect sizes (e.g. Cohen's *d*, Pearson's *r*), indicating how they were calculated

Our web collection on statistics for biologists contains articles on many of the points above.

Software and code

Policy information about availability of computer code

Data collection	No software was used for data collection. We used the 'Palette Cam' app (Alexander Mathers, App Store) to score LAMP images.
Data analysis	Microsoft Excel was used for data analysis.

For manuscripts utilizing custom algorithms or software that are central to the research but not yet described in published literature, software must be made available to editors and reviewers. We strongly encourage code deposition in a community repository (e.g. GitHub). See the Nature Research guidelines for submitting code & software for further information.

Data

Policy information about availability of data

All manuscripts must include a data availability statement. This statement should provide the following information, where applicable:

- Accession codes, unique identifiers, or web links for publicly available datasets
- A list of figures that have associated raw data
- A description of any restrictions on data availability

Data available on request from the authors. Source data are provided with this paper. The images of colorimetric assays are available on the Dryad repository (https://datadryad.org/stash/dataset/doi:10.5061/dryad.2rbnzs7mk).

Field-specific reporting

Life sciences study design

All studies must disclose on these points even when the disclosure is negative.

Sample size	To more accurately determine the number of false positives we aimed to have more than 200 individual negative samples as has been also suggested by one reviewer. A limited number of positive samples was available for the experiments.
Data exclusions	no exclusion
Replication	Replication of findings by two different primer combinations (N gene assay and Orf1a gene assay). Agreement of N gene assay and Orf1a gene assay for tested samples is shown in Supplementary Tables 3 and 4.
Randomization	Randomization is not relevant for this study, since both positive and negative samples undergo the same experimental procedure.
Blinding	Blinding for 'INSTAND' round robin test, Blinding for experiment described in Supp. Fig. 8. Experimentators were blinded for data collection for all experiments.

Reporting for specific materials, systems and methods

We require information from authors about some types of materials, experimental systems and methods used in many studies. Here, indicate whether each material, system or method listed is relevant to your study. If you are not sure if a list item applies to your research, read the appropriate section before selecting a response.

Materials & experimental systems

n/a	Involved in the study	
☒	☐	Antibodies
☒	☐	Eukaryotic cell lines
☒	☐	Palaeontology and archaeology
☒	☐	Animals and other organisms
☐	☒	Human research participants
☒	☐	Clinical data
☒	☐	Dual use research of concern

Methods

n/a	Involved in the study	
☒	☐	ChIP-seq
☒	☐	Flow cytometry
☒	☐	MRI-based neuroimaging

Human research participants

Policy information about studies involving human research participants

Population characteristics	Hospital patients and elderly nursing home inhabitants and employees. We do not have data about population characteristics like gender and age due to privacy protection regulations. Population characteristics should not affect the efficacy of the method.
Recruitment	All individuals included in this study were asked for their voluntary assistance to participate and each individual gave written informed consent before entry into the study. Any potential selection bias should not affect the efficacy of the method.
Ethics oversight	The study was approved by the Ethics Committee of the Saxonian medical chamber (EK-allg-37/10-1). All procedures utilized in this study are in agreement with the 1975 Declaration of Helsinki.

Note that full information on the approval of the study protocol must also be provided in the manuscript.
